# Identification of the Rice Wines with Different Marked Ages by Electronic Nose Coupled with Smartphone and Cloud Storage Platform

**DOI:** 10.3390/s17112500

**Published:** 2017-10-31

**Authors:** Zhebo Wei, Xize Xiao, Jun Wang, Hui Wang

**Affiliations:** Department of Biosystems Engineering, Zhejiang University, 866 Yuhangtang Road, Hangzhou 310058, China; weizhb@zju.edu.cn (Z.W.); wjzyzwz@163.com (X.X.); huiwang@zju.edu.cn (H.W.)

**Keywords:** rice wine, marked age, Smartphone, electronic nose

## Abstract

In this study, a portable electronic nose (E-nose) was self-developed to identify rice wines with different marked ages—all the operations of the E-nose were controlled by a special Smartphone Application. The sensor array of the E-nose was comprised of 12 MOS sensors and the obtained response values were transmitted to the Smartphone thorough a wireless communication module. Then, Aliyun worked as a cloud storage platform for the storage of responses and identification models. The measurement of the E-nose was composed of the taste information obtained phase (TIOP) and the aftertaste information obtained phase (AIOP). The area feature data obtained from the TIOP and the feature data obtained from the TIOP-AIOP were applied to identify rice wines by using pattern recognition methods. Principal component analysis (PCA), locally linear embedding (LLE) and linear discriminant analysis (LDA) were applied for the classification of those wine samples. LDA based on the area feature data obtained from the TIOP-AIOP proved a powerful tool and showed the best classification results. Partial least-squares regression (PLSR) and support vector machine (SVM) were applied for the predictions of marked ages and SVM (R^2^ = 0.9942) worked much better than PLSR.

## 1. Introduction

Chinese rice wine, with its bright brown color and subtle sweet flavor, is a famous ancient wine with a history of 2000 years. Chinese rice wines are fermented from high-quality glutinous rice and wheat. Glutinous rice contains high protein and low fat and the wheat can offer abundant carbon and nitrogen which is a microelement source for the mold and yeast used for fermentation [1]. Therefore, Chinese rice wine is widely known for its health enhancing properties [2]. 

Chinese rice wines with different ageing times often present peculiar flavor features, which is a major determinant of consumer preference and acceptance of rice wines and is also one of the primary characteristics for the pricing of rice wines. In the past, many methods have been performed to detect the flavor features of wines. Traditionally, the determination of the flavor features of rice wines are based on a sensory panel [3]. However, this method depends on the daily physical and mental conditions of the experts and it also requires some expertise. Expensive instruments such as spectrophotometer [4], gas chromatography mass spectrometer [5] and high-performance liquid chromatography [6] have been applied to detect those volatile organic compounds (ethyl acetate, ethyl lactate, isobutanol, isoamyl alcohol, furfural, etc.) that have a relationship with the flavor of rice wines. Because of complex preparation procedures, those instruments cannot be applied for real time or on-line analysis. Additionally, the method also has significant limitations: the flavor substances of the rice wines could not be detected completely by those instruments, therefore the flavor sense of humans could not be accurately reproduced based on measurements taken by these instruments. Thus, it is necessary to develop an on-site method that can express the flavor itself, conveniently and quickly. 

The electronic nose (E-nose) system can closely mimic the organization of human olfactory bulbs, with the application of pattern recognition tools [7,8,9,10]. E-nose does not work in the same way as the precision instruments which evaluate the sample quality just by measuring the concentration of one certain chemical substance—it can obtain global olfactory information from samples through the “soft” measurement of volatile organic compounds (VOCs). Global information can be taken as the ‘fingerprint’ of VOCs, which can be applied for classification and prediction based on appropriate pattern recognition methods. Because of the rapid and low-cost features, E-noses are now widely applied to various food products analysis such as wine [11,12,13,14], other beverages [15,16], food [17,18,19] and fruits [20,21]. In recent years, the development of the E-nose has been driven by the need to design a convenient, portable and specialized system that is available for customized detection. However, most commercial E-noses do not meet the on-site requirements of the current market because of the big volume and computer dependence. Moreover, the concentration and types of VOCs are very different among food products but the sensor array of those commercial E-noses is for general applicability, which can decrease the detection precision.

Smartphones are portable, widely available, user-friendly and therefore well suited to act as an effective platform for on-site detection. At present, Smartphones equipped with different types of sensors can be applied as cost effective, portable investigation tools, especially when applied in locations with limited resources [22,23,24,25]. Currently, Smartphones play important roles in bacteria detection [26], colorimetric detection [27] and genetic analysis [28]. In addition, Smartphones can wirelessly integrate the Internet, establishing the Smartphone as a ubiquitous platform for developing low-cost, easy-to-use and portable instruments. 

In this study, a Smartphone E-nose based on a cloud platform was self-developed at Zhejiang University for identifying rice wines with different marked ages. The E-nose system was controlled by a Smartphone with a specialized application (app) and it connected to a web service infrastructure via the IEEE 802.11 (WiFi). The sampling data obtained by the E-nose was transmitted to the cloud platform through the Smartphone and the results of classification can be made available on the Smartphone. The sensor array of the E-nose is comprised of twelve sensors that have high sensitivity to the VOCs of rice wines and both the “taste and aftertaste information” of the rice wines was obtained by the sensor array. In this study, the customized E-nose was applied for the on-site detection of rice wines with different wine ages and the working efficiency of different pattern recognition methods for classification and prediction was investigated. 

## 2. Materials and Methods

### 2.1. Rice Wine Samples

Five types of rice wines aged 3, 5, 10, 15 and 20 years were tested in this study. All samples were provided by Guyuelongshan Company (Shaoxing city (120°58′ E, 30°01′ N), China, GY-3Y, GY-5Y, GY-10Y, GY-15Y and GY-20Y). A total of 250 bottles of rice wine (50 bottles for each type of rice wine) were random selected during the measurement. All samples were brewed in April 2017 and all the rice wine samples were kept at ambient temperature (25 ± 1 °C) overnight before the analyses were performed.

### 2.2. The Smartphone Electronic Nose Based on Cloud Platform

**Figure 1:**
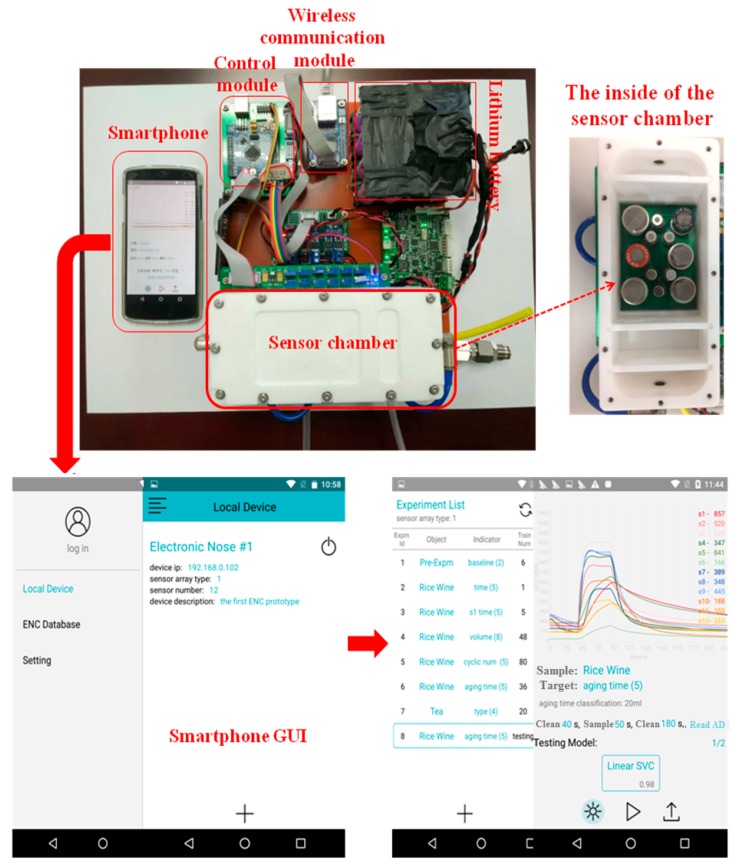
The set-up of the Smartphone electronic nose.

The Smartphone electronic nose applied in the experiment was developed by the agricultural equipment and intelligent detection (AE & ID) team of Zhejiang University and the sensor array of the E-nose was composed of 12 MOS sensors (TGS826 (S1), TGS822 (S2), TGS816 (S3), TGS813 (S4), MQ137 (S5) and MQ138 (S6) TGS2620 (S7), TGS2611 (S8), TGS2610 (S9), TGS2603 (S10), TGS2602 (S11) and TGS2600 (S12)). Table 1 lists the information of the 12 MOS sensors provided by the manufacturer. As shown in Figure 1, the electronic nose mainly included six parts: sensor chamber, control module, power module, communication module, human-computer interface and cloud storage platform (we bought the storage spaces from network operator and the virtual storage space cannot be presented as material object). 12 MOS sensors were placed in a rectangular chamber made of Teflon material and the configuration of those sensors was designed by ANSYS Fluent Software (ANSYS, Inc., Canonsburg, PA, USA) to ensure each sensor can adsorb the target gas efficiently. The MCU STM32F407 was the core unit of the control module which in charge of the sampling parameters setting (gas pump rate, sampling time, clean time, sampling number), solenoid valve switching and sampling model choice (automatic or manual). The power module was composed by 12 lithium batteries which provide the power for controller, gas sensor, sampling pump and solenoid valve. The heating circuit and signal circuit of the gas sensors were separately fed for better activating the gas sensor. The communication module included the wireless module and wireless router and one Smartphone can receive the response signals obtained by E-nose from samples by wireless communication. One special Smartphone application (APP, just for the Android phone at present) was compiled and then installed in a Smartphone that worked as the human-computer interface. The procedures of the E-nose operation, working parameters setting (sampling time, clean time, sampling number), response signals presentation and uploading to the cloud storage were all operated through the APP. The Aliyun (Alibaba Inc., Hangzhou, China) cloud storage space was rented and applied as the cloud storage platform for storing the E-nose response and the identification models of the samples. 

### 2.3. The Investigation of the Rice Wines with the Electronic Nose

The investigation of the rice wines with different marked ages was performed using the E-nose. Each 80 mL sample was poured into a gas blanketing bottle (250 mL). The in-let and out-let of the gas blanketing bottle connected with the out-let and in-let of the sensor chamber, respectively, then the VOCs of the rice wines can circle in the closed-loop sampling channel for the detection without using plastic wrap. Because rice wines are rich in VOCs 10 min is enough for the headspace-generation time. The measurement procedures comprised of three phases: the taste information obtained phase (50 s), the aftertaste information obtained phase (180 s) and the cleaning phase (40 s). At beginning of the taste information obtained phase, the headspace VOCs was pumped into the sensor chamber at a constant rate of 300 mL min^−1^. Then, the response signals increased for the MOS sensors adsorbed VOCs continuously and the responses stabilized after approximately 50 s. The following aftertaste information obtained phase was applied to test the adsorb ability of the sensors to the VOCs using a slight cleaning process (the constant rate of cleaning pump was 100 mL min^−1^). When the VOCs were cleaned gradually away from MOS surface, the conductance decreased following the slight cleaning time which was 180 s. At the cleaning phase, all the sensors were cleaned to their baseline by air with the constant pumping rate of 600 mL min^−1^. The response data, just the taste and the aftertaste information of the wine samples, was transported to the Ali cloud platform through a Smartphone for the later analysis. 

### 2.4. Data Processing

The original responses were preprocessed using the following procedures: firstly, the area method was applied for extracting the feature data from the original responses; then, those feature data were normalized and reduced to two dimensions by different dimensional reduction methods; thirdly, the efficiency of dimensional reduction methods was compared with each other by using the classification plots; finally, the data compressed by the best dimensional reduction method were applied as the input data of the prediction models for the wine age prediction. In the paper, normalized process, PCA and LDA was performed by using SAS v8 (SAS Institute, Cary, NC, USA), the area method, PLSR and SVM were performed by using MATLAB 7.0 (The Math-Works Inc., Natick, MA, USA). 

Principal components analysis (PCA) explains the maximum variance of the data set with the least number of major components without significant loss of information [29]. The explanation of the maximum information of the samples is presented in the form of scores which were applied to detect sample patterns, groupings and similarities or differences between the substrates evaluated [30].

Locally linear embedding (LLE) is a well-known algorithm for non-linear dimensionality reduction. Based on the LLE [31], each sample point in the space can be expressed by a weight matrix in the high-dimensional space, which provides the description of the data points in the low-dimensional space [32]. 

Due to its simplicity and efficiency, linear discriminant analysis (LDA) is one of the most widely-used supervised classification methods [33]. LDA can best separate the classes in the training group based on linear functions which are found by maximizing the ratio of the between-group variance to the within-group variance. LDA has attracted great attention in a number of recognition fields [34].

Partial least squares regression (PLSR) analysis can be used for modeling one or more response variables (Y, such as quality, quantity, pH, ages, etc.) with several predictor variables (X, such as sensor signals, pixel points, wavelength, etc.), through finding a linear regression model by projecting the predicted variables and the observable variables to a new space [35]. 

Support vector machine (SVM) is one of these promising and attractive techniques for pattern classification and regression problems [36]. Different classes of data using SVM coupled with kernel function can be achieved by mapping the original data points from low dimensional space into higher dimensional space [37]. 

## 3. Results and Discussion

### 3.1. Responses Obtained by Electronic Nose from Rice Wines

**Figure 2:**
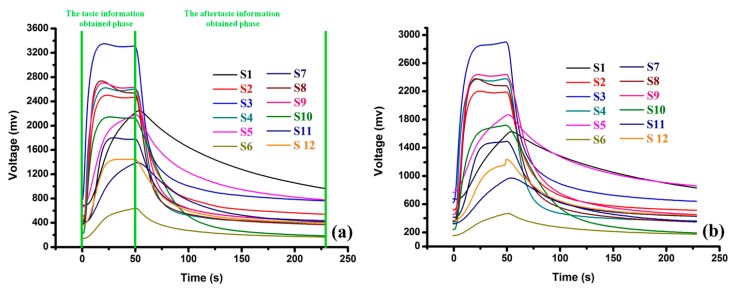
The response signals obtained by the E-nose from the wine samples of 5Y (**a**) and 20Y (**b**).

The typical E-nose response curves obtained from rice wines aged 5 and 20 years are shown in Figure 2. The E-nose coupled with headspace sampling method was applied for the measurement. During the headspace process, the wine sample stood for a while after being poured into the gas blanketing bottle, then the VOCs of the wine sample were enriched in the headspace of the gas blanketing bottle and eventually reached a homogeneous and saturated balance. The interval between the wine sample being poured into the gas blanketing bottle and the VOCs of the wine sample reaching a homogeneous balance was defined as the headspace time. Although the headspace time was just 10 min, the signals of most sensors had already reached their maximums. Rice wines contain many types of VOCs (such as alcohols, aldehydes, esters, etc.), which can fast approach a saturation point in the headspace of the gas blanketing bottle. Then, the signals decreased a little except the signals obtained by S1, S5, S6 and S11, which increased continuously till the end of the taste information obtained phase (50 s) in Figure 2a,b. All the signals decreased smooth and stabilized approximately at the 230th s in the aftertaste information obtained phase. The adsorption-desorption abilities of the sensor array to the rice wines were tested in the measurement and the values obtained from the desorption process which was taken as the aftertaste signals can enrich the olfactory information of the wine samples.

The marked ages can be taken as the presentation of the global quality of rice wines. In this section, the global olfactory information of the rice wines with different marked ages was applied for the further classification and prediction analysis.

### 3.2. Feature Data Extraction of the Original Response

#### 3.2.1. Area Method

**Figure 3:**
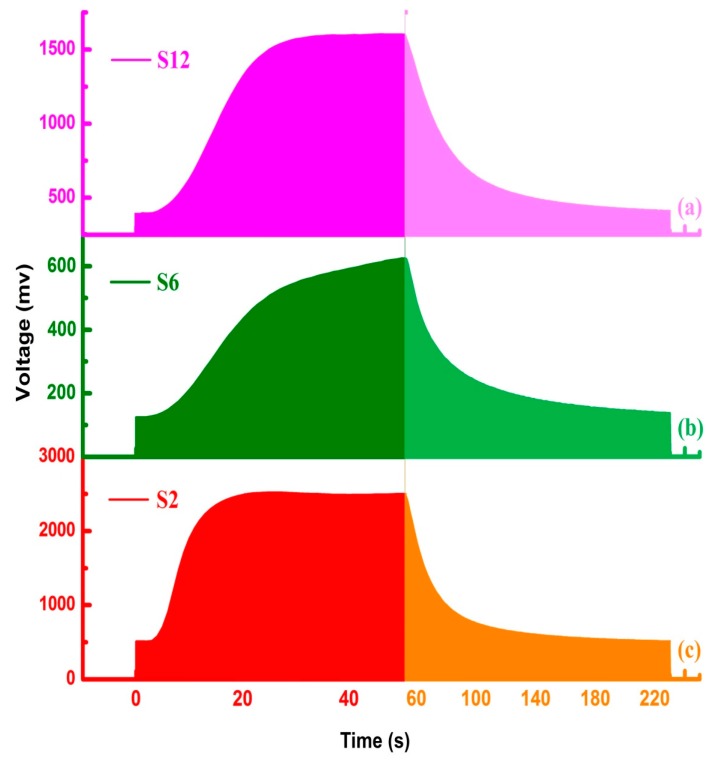
The areas under the response signals obtained by S12 (**a**) S6 (**b**) and S2 (**c**) from the wine samples of 3Y.

In past studies, one certain signal obtained in the stable response phase of E-nose was always taken as the feature data such as the maximum response values, the average value, the maximum differential value, the maximum integral value, the maximum slope value and the time taken to reach the maximum, etc. One piece of certain data is not enough for characterizing the response curve and the most useful information was abandoned during the feature data extraction process. According to the characteristics of the E-nose response curves, the areas under the response curves were calculated as the feature data for the further analysis. As shown in Figure 3, the area method was illustrated by using response curves obtained by S2, S6 and S12 from the rice wines of 3 years. Two types of areas were extracted from the original data: (1) the area just obtained in the taste information obtained phase, where the sum of the areas under the response curves obtained between the 0th s and 50th s; (2) the area obtained in both taste and aftertaste information obtained phase, where the sum of the areas under the response curves obtained between 0 s and 230 s. The classification results based on the two types of feature data set were compared with each other and the better one would be taken for the wine age prediction analysis. 

#### 3.2.2. The Compressed Data Obtained by PCA

The sensor array of the E-nose was composed of 12 MOS sensors and 12 response curves can be obtained in one measurement. The response values of the 12 MOS sensors were the depended variables of time and 12 dimensional data over time were obtained during the measurement. In the study, the twelve-dimensional original data were compressed to the two-dimensional and three-dimensional data and the trajectory of the compressed data in the space can present some information of the wine ages. The compressed process can be expressed as follows: 

The original data obtained by the twelve sensors were

(1) $$X={\left(\vec{x_{1}},\vec{x_{2}},\ldots ,\vec{x_{N}}\right)}^{T}$$

where $\vec{x_{i}}$ is the response of the *i*-th sensor, N = 12.

Then, the original data was processed by the group mean centered model

(2) $$\vec{x_{i}}=\vec{x_{i}}-\frac{1}{N}\overset{N}{\underset{j\;=\;1}{\sum }}\vec{x_{j}}$$

and the covariance matrix ${\mathrm{XX}}^{T}$ was calculated. 

Based on the eigenvalue decomposition of the covariance matrix, the eigenvectors corresponding to the top three greatest eigenvalues were chose to construct the projection matrix:

$$W={\left(\vec{w_{1}},\;\vec{\;w_{2}},\;\vec{{\;w}_{3}}\right)}^{T}$$

This is also the three-dimensional data projected from the twelve-dimensional original data.

**Figure 4:**
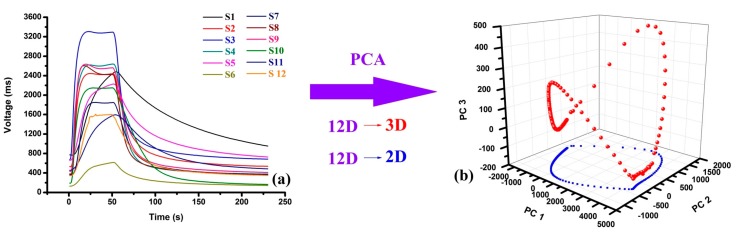
The compression process of the original response by principal components analysis (PCA): (**a**) the original signals, (**b**) the compressed signals.

As shown in Figure 4a, the responses obtained by E-nose from the rice wine of 3Y were recorded as D_230 × 12_. After compressed by PCA, the first 3 PCs were selected for the high accumulative contribution (Figure 4b). Therefore, the original curve D_230 × 12_ was transformed into D_230 × 3_ and D_230 × 2_ and the 3D and 2D projection trajectory of D_230 × 3_ and D_230 × 2_ kept the most feature information of the original trajectory of D_230 × 12_ in the 3D and 2D projection space, respectively. The 3D projection trajectory looked like a saddle and the 2D projection trajectory was an ellipse-like curve, both of the projection trajectories was enclosed.

### 3.3. The Classification of the Rice Wines of Different Marked Ages 

#### 3.3.1. The Classification of the Rice Wine Samples by Using PCA, LLE and LDA Based on Area Feature Data

**Figure 5:**
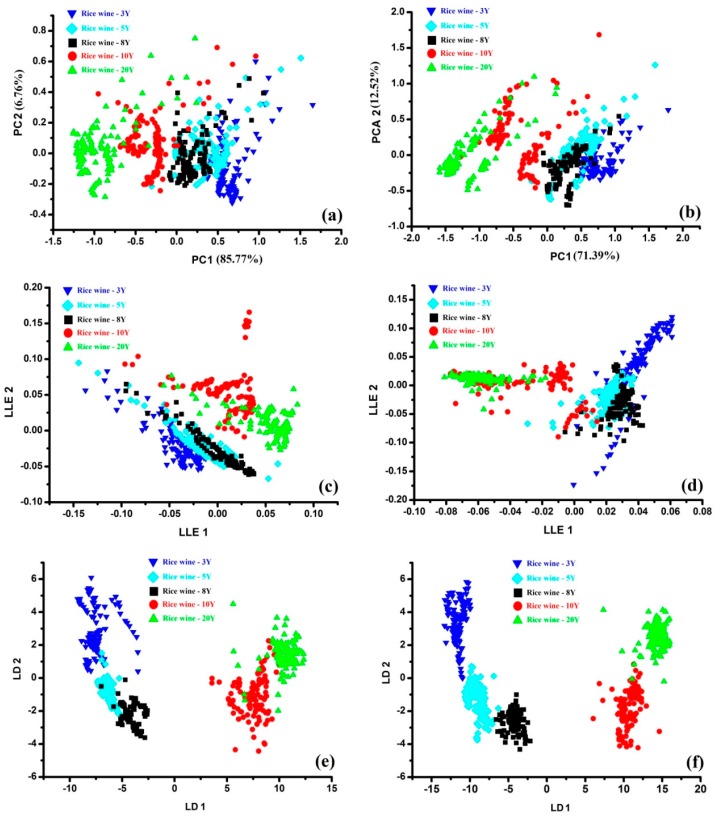
The classification results of PCA based on TIOP (**a**), TIOP-AIOP (**b**). The classification results of LLE based on TIOP (**c**), TIOP-AIOP (**d**). The classification results of LDA based on TIOP (**e**), TIOP-AIOP (**f**).

The PCA score plots for the area feature data obtained by the E-nose are shown in Figure 5a,b. As shown in Figure 5a, the first two PCs (PC_1_ vs. PC_2_) together explained 92.53% of the variance. The separation of the samples was not clearly and each type of the samples cannot be classified from each other obviously. However, the scattered points of each type of wine sample also presented same regulars in the score plot: the wine ages decreased alone the X axis from right to left based on the area data set obtained in TIOP (Figure 5a). The separation of the samples was still not clear enough based on the area data set obtained in TIOP-AIOP (PC_1_ vs. PC_2_ explained 83.91% of the variance), but the scattered points of the samples presented similar location as that in Figure 5a. Those samples can be separated into two parts: I, the 3Y, 5Y and 8Y samples; II, the 10Y and 20Y samples (Figure 5b). The additional aftertaste information enriched the quality information of wine samples, which improved the separation results. Compared with the PCA, LLE did not present more power in the classification analysis nether based on the data set obtained in TIOP or based on the data set obtained in TIOP-AIOP. All the samples can be separated into two parts with or without aftertaste information in the score plots, but the locations of wine samples in LLE plot were still chaos and most samples were over lapped with each other (Figure 5c,d). It was obvious that the classification results based on LDA were the best (Figure 5e,f). Each type of wine sample located regularly in the score plots based on the data set obtained in TIOP and the samples of the same wines grouped together clearly (Figure 5e). The additional aftertaste information improved the classification results obviously: the 3Y, 10Y, 20Y samples grouped more closely and all the samples can be separated with each clearly except two samples of 20Y. 

Above all, LDA was the most powerful tool for the classification of the rice wine samples with different marked ages. It was not only presented a clear classification results, but also showed the regular location of those sample points and the classification result was improved after the addition of aftertaste information. Therefore, the area data set obtained in TIOP-AIOP dimension-reduced by LDA was applied as the input data of the prediction model marked ages of rice wines.

#### 3.3.2. The Classification of the Rice Wine Samples by Using PCA Compressed Feature Data

**Figure 6:**
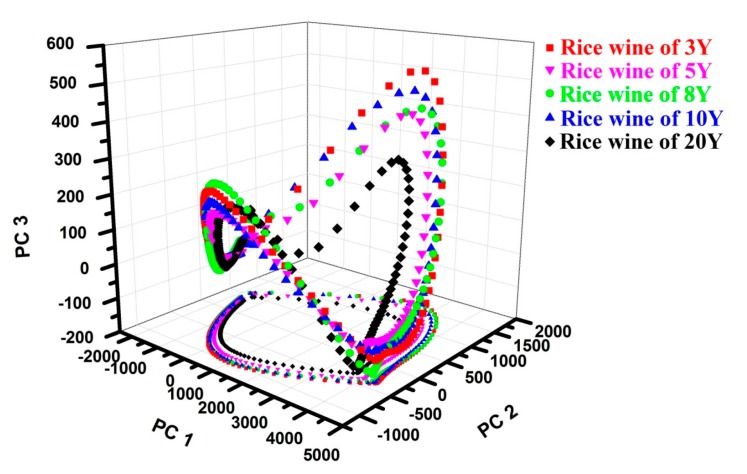
Classification results based on the compressed signal values.

The responses obtained by E-nose from one sample of 3Y, 5Y, 8Y, 10Y and 20Y samples were compressed by PCA, respectively and the projection trajectory of the compressed responses in the 3D and 2D space are shown in Figure 6. In can be seen that the surface area of the 3D projection trajectory of the rice wine of 20Y was the smallest one and that of the rice wine of 3Y was the biggest one. The 3D projection trajectories of 3Y, 5Y and 20Y responses can be clear separated with each other. The 3D projection trajectories of the rice wine of 8Y and 10Y located closely and the projection trajectory area of the rice wine of 8Y was little bigger. The similar regular was also presented in the 2D projection trajectories: the smaller projection trajectory area the longer aging time of rice wine. The differences of the 2D projection trajectories was that the 2D projection trajectories of 3Y, 5Y and 8Y responses located closely with each other and the 2D projection trajectories of 10Y and 20Y responses were separated clearly with each other. The regularities presented by the configuration of 2D projection trajectories were similar as that presented in the classification results using PCA (Figure 5b). Therefore, the marked ages of rice wines also can be qualitative identified by the areas of the 2D and 3D projection trajectories. 

#### 3.3.3. The Prediction of Marked Ages of the Rice Wine Samples

PLSR and SVM were applied for the prediction of wine ages. 250 samples (50 samples of each type of Chinese rice wine) were chosen of which 150 samples (30 samples of each type) were applied as the training set and 100 samples (20 samples of each type) were applied as a testing set. 

**Figure 7:**
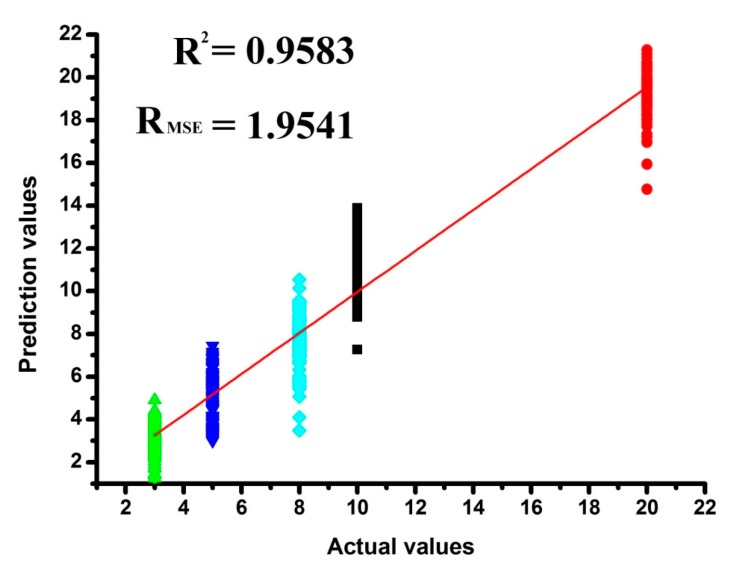
The prediction results for rice wines with different marked ages based on PLSR.

Based on the area data set obtained in TIOP-AIOP dimension-reduced by LDA, the PLSR prediction results for marked ages are presented in scatter plot (Figure 7). The ordinate and abscissa axes represent the predicted and actual values of the marked ages, respectively. Although the correlations between measured and predicted values of marked ages was over 0.95 (R^2^ = 0.9583), however, the root mean square error (R_MSE_) of the prediction values was big (R_MSE_ = 1.9541, even though *p*-value was lower than 0.05). It also can be seen from Figure 7 that the point of each type of wine was scattered seriously: the differences between the upper and lower deviation of the prediction values of 3Y and 5Y were almost 5 years, the differences between the upper and lower deviation of the prediction values of 8Y, 10Y and 5Y were almost 10 years. Therefore, the PLSR prediction result was not a good one. 

**Figure 8:**
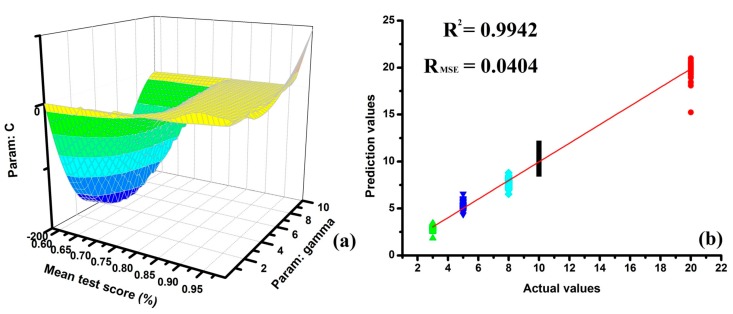
The prediction results for rice wines with different marked ages based on SVM: (**a**) contour plots for the prediction parameters, (**b**) the prediction results based on SVM.

The radial basis function (RBF) was applied as the core function of the SVM methods to predict wine ages. Grid searches were performed with parameters error-surface based on leave-one-out cross validation procedure. The parameters could be tuned simultaneously and those ones with the lowest error in the smooth area would be selected for building the most robust model. The contour plots of the optimization the parameters for the prediction of wine ages of the rice wines are shown in Figure 8a. After automatic test, γ = 0.5963, C = 8.6851 and best score = 0.995 were picked out as the best parameters for predicting wine ages. The correlation between predicted and real wine age are shown in Figure 8b and the prediction results of SVM showed a clear indication of the E-nose ability. The SVM can clear notice a positive trend in the prediction of marked ages and the prediction results of SVM were R^2^ = 0.9942 and R_MSE_ = 0.0404. The average difference values between the upper and lower deviation of the prediction values was almost 3 years and the prediction results of SVM were much better that of PLSR. Therefore, the E-nose coupled with SVM was proved a powerful tool for the identification of the wine ages of rice wines. 

## 4. Conclusions

In this study, the E-nose was developed for the identification of rice wines with different marked ages. A Smartphone was installed with a special app that worked as the human-computer interface and the signals obtained by the E-nose were stored in the cloud storage platform through the Smartphone. The feature data were extracted using the area method and then processed by PCA, LLE and LDA for classification and processed by PLSR and SVM for prediction. The main results were as follows:

(1) The communication between the Smartphone and sensor array was a wireless module and the Smartphone worked as a computer and the Aliyun storage platform worked as hard disc. All the response signals and identification models was stored in the Aliyun through the Smartphone. Compared with the traditional E-nose, the E-nose developed in this study saved lots of space. Moreover, the E-nose can be applied for on-line detection because its small size and portability.
(2) The measurement of the E-nose was separated into two phases: TIOP and AIOP and the aftertaste information was obtained in the AIOP. The results of PCA, LLE and LDA demonstrated that the addition of aftertaste information has a positive effect on the classification and the LDA, based on the feature data obtained from TIOP-AIOP, provided the best identification results. SVM worked more efficiently than PLSR for prediction and it showed the higher correlation (R^2^ = 0.9942) and the lower root mean square error (R_MSE_ = 0.0404).

The E-nose proved to be a powerful tool for identifying rice wines with different marked ages. At present, all the samples were detected in the lab, with a stable environment meaning that the temperature effect on the E-nose system could be ignored. The E-nose system will be improved by addition of a temperature control module for controlling the sampling gas temperature, which can make sure the on-line monitoring work of the E-nose will not be affected by the complex environment in the wine factory.

## Figures and Tables

**Table 1:** The information of the 12 MOS sensors.

Sensors Labels	Sensor Name	Target Gases	Typical Detection Range
S1	TGS826	Ammonia	30–300 ppm
S2	TGS822	Alcohol, Solvent vapors	50–5000 ppm
S3	TGS816	Methane, Butane, Propane	500–10,000 ppm
S4	TGS813	Methane, Butane, Propane	500–10,000 ppm
S5	MQ138	Aldehydes, alcohols, ketones, aromatics	1 to 100 ppm
S6	MQ137	Ammonia	5–500 ppm
S7	TGS2620	Alcohol, Solvent vapors	50–5000 ppm
S8	TGS2611	Methane	1–25%
S9	TGS2610	Butane, Propane	1–25%
S10	TGS2603	Trimethylamine, methyl mercaptan	1–10 ppm
S11	TGS2602	VOCs, ammonia, hydrogen sulfide	1–30 ppm
S12	TGS2600	hydrogen, ethanol	1–30 ppm

## Supplementary Materials

Supplementary File 1
