# Supplementary material for: Identification of the Rice Wines with Different Marked Ages by Electronic Nose Coupled with Smartphone and Cloud Storage Platform
Source: Sensors (Basel). 2017 Oct 31;17(11):2500. doi: 10.3390/s17112500 (PMC5712832; doi:10.3390/s17112500)
Supplement: Supplementary File 1 [file sensors-17-02500-s001.pdf]

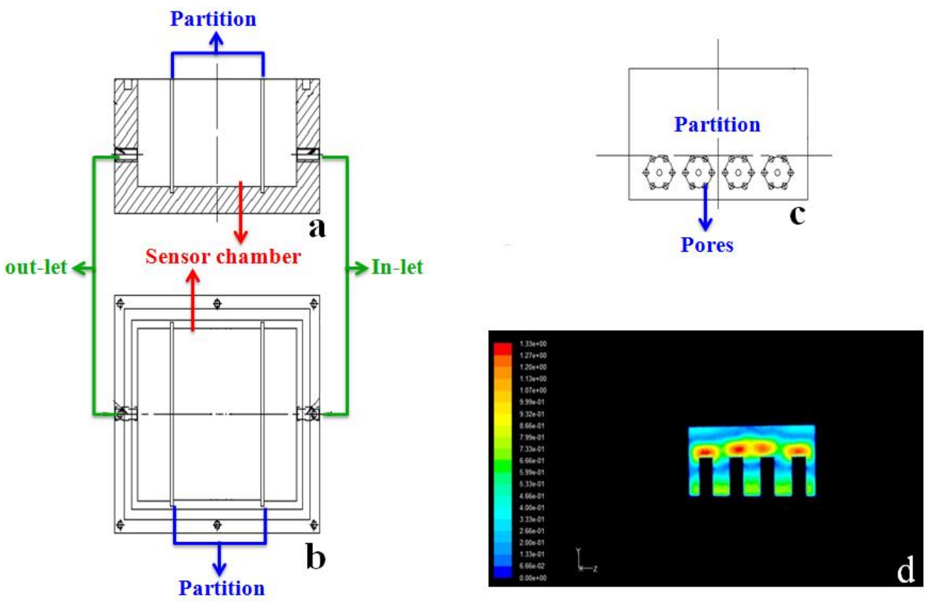

Fig. S1 The profile (a) and top view (b) of the chamber. The room of the chamber was separated into 3 parts by two partitions (c), and the sensor array is put into the middle room of the chamber. After optimized by ANSYS Fluent Software, the configuration and location of the pores in the partitions, the location of the in-let and out let was optimized. The pressure gradients presented that the airflow was homogeneous when got through each sensor (d)

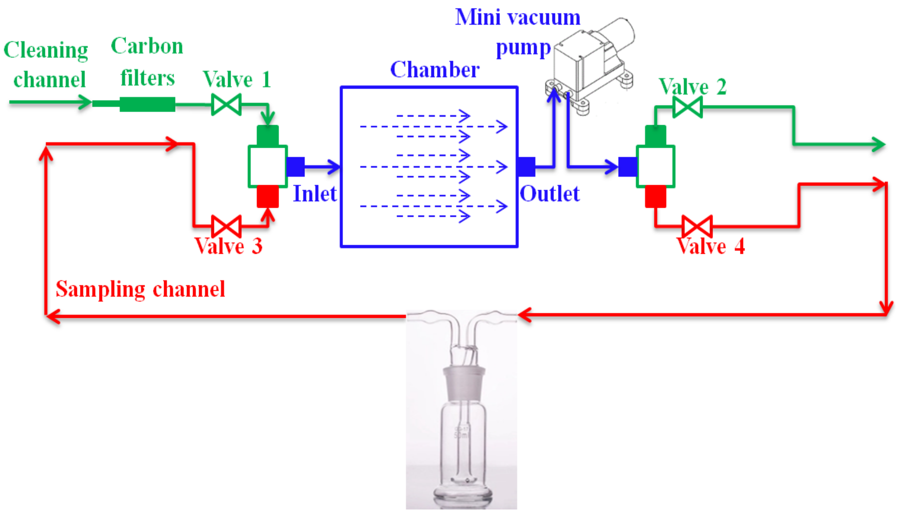

Fig. S2 The valve 1 and valve 2 are closed during the sampling phase, and the valve 3 and valve 4 are closed during the cleaning phase. A carbon filter was added in the cleaning channel to clean the inlet airflow.
